# Comfort Always: The Importance of Providing Psychological Support to Neurology Staff, Patients, and Families During COVID-19

**DOI:** 10.3389/fpsyg.2020.573296

**Published:** 2020-10-22

**Authors:** Jennifer A. Foley, Edgar Chan, Natasja van Harskamp, Lisa Cipolotti

**Affiliations:** Department of Neuropsychology, National Hospital for Neurology and Neurosurgery, London, United Kingdom

**Keywords:** neuropsychology, COVID-19, healthcare workforce, mental health, patients and caregivers

## Abstract

**Background:**

Although the impact of COVID-19 disruption on healthcare staff is increasingly understood, there has been no discussion of how it affects neurological patients and their families. This study sought to understand the impact of COVID-19 on staff, patients and families.

**Methods:**

The Department of Neuropsychology at the National Hospital for Neurology and Neurosurgery established three new support services for staff, patients and families. Semi-structured interviews elicited concerns and if these were affected by COVID-19. Staff members were asked to complete the General Health Questionnaire-12.

**Results:**

Few staff members presented for support, but nearly all indicated significant distress, reflecting increased anxiety and reduced social support. Patients described exacerbated emotional, cognitive and physical concerns, and greater vulnerability to isolation and economic hardship. Families and carers reported increased distress arising from hospital lockdown.

**Conclusion:**

COVID-19 disruption affects staff, patients and families. Patients and families described additional challenges, which emphasize the importance of providing psychological support during these extraordinary times.

## Introduction

To meet the challenges posed by the COVID-19 emergency, health services have had to transform radically. Many clinicians have been redeployed to the frontline and/or temporary new hospitals. Clinical academics have been asked to return to clinical duties. Specialist services must now provide much more general medicine. As health services scramble to cope with the influx of COVID-19 patients, services for other patient groups, including those with neurological conditions, have necessarily been curtailed. Even when current arrangements are stepped down, it is likely that governmental recommendations for social distancing, case isolation and vulnerable patient shielding will continue to limit service delivery. Furthermore, patients are also less willing to use the limited clinical services that are available, as evidenced by the marked decrease in stroke admissions (Markus and Brainin, 2020). The full implications of these changes for neurological patients have yet to be fully realized. Indeed, although there is increased understanding of the psychological implications of COVID-19 on healthcare staff (Gold, 2020; Zhu et al., 2020), and of the neurological and psychiatric manifestations of COVID-19 (Manji et al., 2020; Rogers et al., 2020), hitherto there has been no discussion on the psychological impact of COVID-19 disruption on neurological patients and their families.

The COVID-19 emergency has caused an “unprecedented level of individual and societal fear and anxiety” (Tsamakis et al., 2020). Its threat, and its constant prominence in the media, has fueled a cataclysm of mental health issues (Garfin et al., 2020), particularly for those with pre-existing vulnerabilities (Gobbi et al., 2020). For patients with neurological conditions, these stressors have been combined with dramatic reductions in clinical care and enforced isolation; potentially having a ruinous effect upon mental health (Helmich and Bloem, 2020; Stojanov et al., 2020; Yao et al., 2020). As neurological patients already carry an increased risk of neuropsychiatric disturbance, the psychological impact may be catastrophic. Despite this, there has been no focus on mental health in any of the existing neurology recommendations (Association of British Neurologists, 2020).

Similarly, there has been no guidance on how best to support neurological patients’ families and/or informal carers (henceforth termed “family members”). Hospital restrictions have prohibited visitors, limiting the education and support family members receive from healthcare professionals, even following acute neurological events. For those supporting outpatients, nationwide restrictions have meant they may now be confined to their caring role without breaks, respite or support (e.g., Edwards and Carroll, 2020). Unfortunately, such inadequate preparation for discharge and insufficient support may well become a lethal cocktail.

In response, we redesigned our clinical services at the Department of Neuropsychology at the National Hospital for Neurology and Neurosurgery (NHNN; Cipolotti et al., 2020; Foley et al., 2020). Before COVID-19, the Department focussed on the assessment, management and treatment of patients with complex neurological, neurosurgical and neuropsychiatric conditions. However, like other services (Coetzer and Bichard, 2020), the emergency has meant that we have had to adapt our usual care. Assessments are now limited to inpatients with acute symptoms or on emergency pathways (e.g., brain tumor, stroke). All therapeutic support to outpatients (e.g., Parkinson’s disease, multiple sclerosis) is now delivered remotely, either by telephone or video. We have also developed three new services. Following best practice guidance (World Health Organization [WHO], 2020) and research emerging from China (Chen et al., 2020), we like many others (e.g., Waldman et al., 2020) have developed new neuropsychological support services for staff. We also developed new services designed to support our neurological patients and their family members. Here, we present our preliminary findings on these new services to illuminate how COVID-19 has impacted staff, patients and families, and provide recommendations for future care.

## Methods

For staff, the new neuropsychological support services for staff consisted of daily telephone and twice-weekly walk-in and telephone clinics, offering one-to-one support. This new service was advertised to all staff (approximately 1,500 clinical and non-clinical staff). All those presenting for support underwent detailed clinical psychological assessment using a semi-structured interview (see Appendix 1), including questions relating to the mental health impact of COVID-19 based on the limited available literature coming from China (for a review, see Rajkumar, 2020). The interview elicited staff members’ concerns; whether these were related to COVID-19; and their history of psychological difficulties. Based upon this and the presenting problems, they were offered follow-up of tailored psychological support; referred to neuropsychiatry or their general practitioner for medication review; or discharged. Staff members were asked to provide demographic information; profession; length of service at NHNN; location of work; and whether they had contact with COVID-19 patients. They also completed the General Health Questionnaire-12 (GHQ; Goldberg and Williams, 2000) to assess the presence of psychological symptoms. A binary scoring method was used, with a total score of 4 or above indicating psychological distress.

For patients, all those who had neuropsychological outpatient appointments rescheduled were offered a telephone consultation, offering one-to-one psychological support. This service was also advertised to NHNN consultants and local community neuropsychology teams. Patients opting in to this new service underwent clinical psychological assessment using a semi-structured interview (see Appendix 2). This sought to elicit their concerns; whether these were affected by COVID-19; and their history of psychological difficulties. Based upon this and the presenting problems, patients were offered follow-up of tailored psychological support or discharged. GHQ scores, patients’ demographic and clinical details were collected.

For family members of inpatients or outpatients, telephone clinics were established and advertised to NHNN clinical teams. Those referred for support underwent clinical psychological assessment using a semi-structured interview (see Appendix 3). This sought to elicit the main concerns; COVID-19-related changes; and history of psychological difficulties Following this, the family member was offered one-to-one psychological support; psychoeducation on the neurological condition, cognitive functioning, mood and fatigue; signposting to sources of further information and support; and/or relevant further guidance, sent through the post. Demographic and clinical details were collected, as well as their relationship to the patient.

Qualitative responses to the semi-structured interviews and concerns noted by the neuropsychologists were transcribed, coded and analyzed using a grounded theory approach (Strauss and Corbin, 1998) to elicit emerging themes. All identified themes were compared within each group (staff, patient or family member) to form overarching categories. Categories identified in earlier sessions were then cross-referenced with those from later sessions to determine when data saturation was sufficient. We illustrate each of these thematic categories with quotations. The service audit was done in compliance with the Helsinki Declaration.

## Results

To date, 23 staff members have presented for psychological support, including two referred by their managers. The majority were female (86.9%), with a mean age of 40 years (range 25–63). Most were clinical staff (82.6%), but professional roles were diverse, ranging from cleaner to consultant. Half worked on inpatient wards (56.5%), and had or were about to have contact with COVID-19 patients (43.5%). Length of service at NHNN ranged from less than 1 to 12 years (mean = 5.75 years). On the GHQ, mean score was 6.56/12, with most (77.8%) scoring at or above clinical cut-off, indicating significant levels of psychological distress. Thematic categories emerging from the structured interviews and illustrative quotations are presented in Table 1a.

**TABLE 1:** Percentage endorsing each identified thematic category emerging from neuropsychological services for (a) staff, (b) patients, and (c) family members.

	**Identified thematic categories**	**Illustrative quotations**	**%**
(a) Staff (*n* = 23)	General anxiety	“It’s an ongoing trauma,” “It feels hard to switch off”	70%
	Loss of social support (e.g., loss of structure at work, loss of social network because of distancing rules)	“I feel alone”	52%
	Concern about infection (of themselves or family members)	“I’m scared I will get COVID-19 and die,” “I’m worried about passing it on to my landlady”	43%
	Concern about redeployment	“I don’t have the skills to work on the ward,” “I feel underqualified”	26%
	Concerns about PPE	“I don’t have the correct PPE”	13%
(b) Patients (*n* = 21)	Emotional challenges (e.g., anxiety, low mood)	“I’m anxious about the future,” “My anxiety has escalated”	81%
	Concerns about cognitive/physical difficulties (e.g., worsening of neurological symptoms, fatigue)	“I’m frustrated by my dystonia,” “I’m worried about my memory”	48%
	Difficulties with isolation (e.g., not being able to receive same care or attend day centers)	“I’m unable to attend my usual activities”	29%
	Financial/work concerns (e.g., redundancy, fewer work opportunities)	“I’m worried I will be laid off,” “My employment opportunities have been decimated”	24%
	Delayed or reduced clinical care (e.g., delayed surgery, reduced rehabilitation)	“I’m not getting adequate care,” “I feel very let-down”	19%
	Concern about infection	“I’m worried my wife will pass the virus onto me,” “I’m vulnerable”	14%
(c) Family members (*n* = 26)	Excluded from patient’s care (e.g., unable to visit, not included in clinical discussions)	“Information seems restricted,” “I don’t know how much to call,” “I feel in limbo”	50%
	Emotional challenges (e.g., shock, anxiety, reduced social support)	“I’m anxious about her coming home,” “I have no support”	46%
	Delayed or reduced clinical care (e.g., slow to present to stroke services, faster discharge despite significant needs)	“There will be no rehabilitation options,” “Delayed appointments mean that his symptoms are getting worse”	42%
	COVID-19 (e.g., bereavements, anxiety about virus transmission)	“She won’t be able to keep to COVID rules,” “I’m worried she’ll get the virus in hospital”	38%
	Difficulties communicating with inpatients (e.g., sensory/cognitive deficits, lack of mobile phone)	“I cannot visit and only have limited time on the phone,” “I’ve had no contact”	38%

The most common theme was raised general anxiety. This manifested as increased worry and panic attacks, with anxiety about themselves, others and/or the future. For nearly all (93.8%), this anxiety was caused or exacerbated by COVID-19. Other frequent themes were loss of social support (e.g., at work and in general because of social distancing rules), concern about infection (with equal numbers describing concern about themselves or friends/family members contracting COVID-19), and work stressors (redeployment and PPE). Half of the staff members (43.4%) revealed previous history of anxiety and/or depression, with most of these (70%) requiring formal psychological intervention in the past. Although those with previous history had higher scores on the GHQ (mean = 7.8) than those without (mean = 4.9), there were no differences in themes raised. Half (56.5%), including all those with previous history of psychological difficulties, were offered follow-up, with two referred onto neuropsychiatry or their general practitioner for consideration of anti-anxiety medication.

Telephone consultations have been held with 21 outpatients with stroke (29%), Parkinson’s disease (14%), multiple sclerosis (14%), epilepsy (10%), neurosurgical conditions (10%), ataxia (0.5%), metabolic disorder (0.5%), dystonia (0.5%), neuro-oncology (0.5%), or memory concerns awaiting assessment/diagnosis (0.5%). Half were male (52%), with an average age of 54 years (range 27–89). On the GHQ, mean score was 6.75/12, with most (75.0%) scoring at or above clinical cut-off, indicating significant levels of psychological distress. Thematic categories emerging from the structured interviews and illustrative quotations are presented in Table 1b.

The most common theme was emotional challenges. This reflected both anxiety and low mood, triggered by the neurological symptoms (88.2%) and further exacerbated by COVID-19 (82.3%). For example, one patient described feeling anxious about coping with declining function caused by ataxia and this was compounded by the additional pressures of managing home-schooling and the threat of redundancy. Other frequent themes included concerns about cognitive/physical difficulties, with nearly half of these distressed about the impact of COVID-19 on their hospital care and/or carer support. Several described difficulties coping with isolation, particularly those with sensory disabilities, no longer able to attend day centers or receive informal care, and/or those with fewer social contacts, unable to ask others for support with essential activities. Several described how the emergency had affected their working ability and financial resources, already hampered by neurological disability. Two thirds (67%) were offered follow-up.

Telephone consultations have been held with 26 family members and carers of patients with stroke (85%) or Parkinson’s disease (15%). 19% also had suspected or confirmed COVID-19. The majority were inpatients (81%), male (76%), and with an average age of 67 years (range 27–90). The relationship to the patient was partner (46%), child (44%), sibling (8%), or parent (4%). Themes emerging from the consultations are presented in Table 1c.

The most common theme was feeling excluded from the patient’s care, mostly arising because they were unable to visit them in the hospital. This was particularly upsetting for those who were spokesperson for a patient with communication and/or cognitive difficulties, with family members worrying they were not receiving appropriate care. For example, one family member was particularly concerned that the staff had not been informed that his father, an inpatient with Parkinson’s disease, needed to receive his Parkinson’s medications on time. Another frequent theme highlighted the unique emotional challenges they were facing; many expressed shock about their family member’s diagnosis and felt unsupported by both hospital staff and their social network. Others described anxiety about their family members’ care and not being able to communicate with them. Many described concerns about the impact of COVID-19 on their family members’ clinical care, describing delayed or reduced services. Some were anxious about expedited discharge and their ability to cope at home with the patient. Others were worried about accessing specialist services after discharge. It is notable that all of these themes were directly affected by COVID-19. A third of all families and carers (31%) were offered follow-up.

## Discussion

These preliminary findings illustrate the psychological impact of COVID-19 on staff, neurological patients and their families. Although few staff members presented for formal psychological support, nearly all endorsed significant levels of distress on the GHQ, with half requiring follow-up within our service and/or referral for medication review. Many had previous mental health history, suggesting that this increased vulnerability to psychological distress during the current emergency. Interestingly, this previous history did not affect the nature of their concerns; nearly all described increased anxiety and many reporting feeling estranged from their normal sources of support. These findings suggest that these issues are universal, but experienced as more challenging by those with previous mental health issues. This highlights the importance of providing formal staff support to those with higher levels of distress and/or previous mental health history. In the future, it may be useful to promote staff support by embedding psychologists within staff teams to facilitate disclosure of psychological distress and provide individually tailored support.

We were also able to document the profound impact of COVID-19 disruption on patients and their families. Patients reported high rates of distress on the GHQ and described how their emotional, cognitive and physical symptoms exacerbated by the emergency. Many also described how their neurological condition exacerbated their vulnerability to loneliness and economic hardship. These findings are particularly important given the expected long-term health service changes and looming economic downturn.

For family members, many concerns were a direct result of not being allowed into hospital, with reports of feeling excluded from patients’ care, and feeling bereft of the usual support provided by hospital staff. These findings illustrate the necessity of family liaison, at admission so they can provide information about patients’ needs and wishes, and throughout, so they can feel involved and supported by healthcare professionals, particularly in preparation for discharge.

These preliminary findings, although limited by small participant numbers, provide a snapshot of the psychological impact of the COVID-19 emergency. Unfortunately, we were not able to provide GHQ scores for family members, but we were able to document that half of these described acute psychological distress. In the future, we would like to compare all groups with the same measures and consider how these and the issues raised change as we emerge from the height of the pandemic to learning to live with its impact.

During this pandemic, we have witnessed the profound physical and psychological impact of the COVID-19 emergency, emphasizing the importance of providing direct psychological care to hospital staff, patients and families. In this study, we have found that although staff, patients and families all demonstrate psychological distress and reduced levels of social support, only patients and families bear the additional burden of neurological illness and disability. This highlights the need of providing psychological support to these vulnerable groups during these extraordinary times.

## Data Availability Statement

The datasets presented in this article are not readily available because: We do not have permission to share these data. Requests to access the datasets should be directed to JF, Jennifer.Foley@nhs.net.

## Ethics Statement

The studies involving human participants were reviewed and approved by the local clinical governance committee of the National Hospital for Neurology and Neurosurgery. Written informed consent for participation was not required for this study in accordance with the national legislation and the institutional requirements.

## Author Contributions

JF, EC, NH, and LC: conception and design of the study, collection and assembly of data, and revising it critically for important intellectual content. JF, EC, and LC: analysis and interpretation of the data and drafting the article. All authors contributed to the article and approved the submitted version.

## Conflict of Interest

The authors declare that the research was conducted in the absence of any commercial or financial relationships that could be construed as a potential conflict of interest.

## Appendix 1: NHNN Staff Support Service Semi-Structured Interview

### NHNN Staff Support Service

1. Main concerns

Getting infected?
Family worried?
Bringing the virus home?
Dealing with uncooperative or panicked patients?
Shortage of PPE?
Feeling incapable of caring for critically ill?
Lack of rest?

2. COVID –related changes (e.g., home environment, transport, isolation, role change)
3. Pre-COVID psychological factors/Risk factors/? E.g., previous/current history of psychological problems, medication use, suicide risk?
4. Protective factors?

**Plan/Outcome:**

□ No Follow-up □ Follow-up □ Refer to Neuropsychiatry/General Practitioner

## Appendix 2: NHNN Patient Support Service Semi-Structured Interview

### NHNN Patient Support Service

1. Main concerns

Health conditions?
Access to medical follow-up?

2. COVID –related changes (e.g., home environment, transport, isolation, role change)
3. Pre-COVID psychological factors/Risk factors/? E.g., previous/current history of psychological problems, medication use, suicide risk?

Previously poor mental health/self-harm?
Separation from family/support networks?
Reliance on social services?
Low household income?
Literacy/access to information?

4. Protective factors?

**Plan/Outcome:**

□ No Follow-up □ Follow-up □ Refer to Neuropsychiatry/General Practitioner

## Appendix 3: NHNN Family Member Support Service Semi-Structured Interview

### NHNN Family Member Support Service

1. Main concerns

Health conditions?
Access to medical follow-up?

2. COVID –related changes (e.g., home environment, transport, isolation, role change)
3. Pre-COVID psychological factors/Risk factors/? E.g., previous/current history of psychological problems, medication use, suicide risk?

Previously poor mental health/self-harm?
Separation from family/support networks?
Reliance on social services?
Low household income?
Literacy/access to information?

4. Protective factors?

**Plan/Outcome:**

□ No Follow-up □ Follow-up □ Refer to Neuropsychiatry/General Practitioner

## References

[B1] Association of British Neurologists (2020). *Guidance on COVID-19 for People With Neurological Conditions, Their Doctors and Carers.* Avaliable at: www.theabn.org/page/covid-19_patients (accessed 20 May 2020).

[B2] Chen Q. Liang M. Li Y. Guo J. Fei D. Wang L. (2020). Mental health care for medical staff in China during the COVID-19 outbreak. *Lancet Psychiat.* 7 e15–e16. 10.1016/S2215-0366(20)30078-X PMC7129426 32085839

[B3] Cipolotti L. Chan E. Murphy P. van Harskamp N. Foley J. A. (2020). Factors contributing to the distress, concerns, and needs of UK Neuroscience health care workers during the COVID-19 pandemic. *Psychol. Psychother.* e12298. 10.1111/papt.12298 32672411 PMC7404511

[B4] Coetzer R. Bichard H. (2020). The challenges and opportunities of delivering clinical neuropsychology services during the Covid-19 crisis of 2020. *Panamer. J. Neuropsychol.* 14 29–34. 10.7714/CNPS/14.1.204

[B5] Edwards E. Carroll C. (2020). In reply to: Helmich and Bloem (2020) “The Impact of the COVID-19 Pandemic on Parkinson’s Disease: hidden sorrows and emerging opportunities”. *J. Park Dis.* 10 1267–1268. 10.3233/JPD-202169 32597820 PMC7458502

[B6] Foley J. A. Chan E. van Harskamp N. Cipolotti L. (2020). Covid-19 emergency: redesigning the department of neuropsychology services at the National Hospital for Neurology and Neurosurgery. *Clin. Psych. Forum.* 3289 8–11.

[B7] Garfin D. R. Silver R. C. Holman E. A. (2020). The novel coronavirus (COVID-2019) outbreak: amplification of public health consequences by media exposure. *Health Psychol.* 39 355–357. 10.1037/hea0000875 32202824 PMC7735659

[B8] Gobbi S. Plomecka M. B. Ashraf Z. Radziński P. Neckels R. Lazzeri S. (2020). Worsening of pre-existing psychiatric conditions during the COVID-19 pandemic. *medRxiv* [Preprint] 10.1101/2020.05.28.20116178 PMC7772353 33391049

[B9] Gold J. A. (2020). Covid-19: adverse mental health outcomes for healthcare workers. *BMJ* 369:m1815. 10.1136/bmj.m1815 32371465

[B10] Goldberg D. Williams P. (2000). *General Health Questionnaire (GHQ).* Swindon: NFER Nelson.

[B11] Helmich R. C. Bloem B. R. (2020). The impact of the COVID-19 pandemic on Parkinson’s disease: hidden sorrows and emerging opportunities. *J. Park Dis.* 10 351–354. 10.3233/jpd-202038 32250324 PMC7242824

[B12] Manji H. Carr A. S. Brownlee W. J. Lunn M. P. (2020). Neurology in the time of COVID-19. *J. Neurol. Neurosurg. Psychiatry.* 91 568–570. 10.1136/jnnp-2020-323414 32312872

[B13] Markus H. S. Brainin M. (2020). COVID-19 and stroke—A global world stroke organization perspective. *Int. J. Stroke* 15 361–364. 32310017 10.1177/1747493020923472 PMC11927026

[B14] Rajkumar R. P. (2020). COVID-19 and mental health: a review of the existing literature. *Asian J. Psychiatry* 52:102066 10.1016/j.ajp.2020.102066Rajkumar PMC7151415 32302935

[B15] Rogers J. P. Chesney E. Oliver D. Pollak T. A. McGuire P. Fusar-Poli P. (2020). Psychiatric and neuropsychiatric presentations associated with severe coronavirus infections: a systematic review and meta-analysis with comparison to the COVID-19 pandemic. *Lancet Psychiat.* 7 611–627. 10.1016/S2215-0366(20)30203-0 PMC7234781 32437679

[B16] Stojanov A. Malobabic M. Milosevic V. Stojanov J. Vojinovic S. Stanojevic G. (2020). Psychological status of patients with relapsing-remitting multiple sclerosis during coronavirus disease-2019 outbreak. *Mult. Scler Relat. Disord.* 45:102407. 10.1016/j.msard.2020.102407 32702641 PMC7365115

[B17] Strauss A. Corbin J. (1998). *Basics of Qualitative Research Techniques.* Thousand Oaks, CA: Sage publications.

[B18] Tsamakis K. Triantafyllis A. S. Tsiptsios D. Spartalis E. Mueller C. Tsamakis C. (2020). COVID-19 related stress exacerbates common physical and mental pathologies and affects treatment. *Exp. Ther. Med.* 20 159–162. 10.3892/etm.2020.8671 32509006 PMC7271730

[B19] Waldman G. Mayeux R. Claassen J. Agarwal S. Willey J. Anderson E. (2020). Preparing a neurology department for SARS-CoV-2 (COVID-19): early experiences at columbia university irving medical center and the New York presbyterian hospital in New York City. *Neurology.* 94 886–891. 10.1212/WNL.0000000000009519 32253352 PMC7963356

[B20] World Health Organization [WHO] (2020). *Mental Health and Psychosocial Considerations During the COVID-19 Outbreak.* Avaliable at: www.who.int/docs/default-source/coronaviruse/mental-health-considerations. pdf (accessed 20 May 2020).

[B21] Yao H. Chen J. H. Xu Y. F. (2020). Patients with mental health disorders in the COVID-19 epidemic. *Lancet Psychiat* 7:e21 10.1016/S2215-0366(20)30090-0 PMC7269717 32199510

[B22] Zhu Z. Xu S. Wang H. Liu Z. Wu J. Li G. (2020). COVID-19 in wuhan: immediate psychological impact on 5062 health workers. *medRxiv* [Preprint] 10.1101/2020.02.20.20025338 PMC7311903 32766545

